# Ricord

**Published:** 1869-01

**Authors:** 


Ricord.—We condense the following account of the famous Syphilographer from the columns of *L'Evenement Medicale:*

Philip Ricord was born in Baltimore, U. S., December 10, 1800. At twenty years of age he went to Paris to complete his education and study law. Having one day accompanied a friend to the Hotel Dieu, he became so enamored of a lecture of Dupuytren, that at once, without consulting his father, he deserted the benches of the School of Law for the amphitheater of the School of Medicine. At the end of three years of diligent study he was appointed interne, and entered the service of Dupuytren.

In 1826, young Ricord received his degree of doctor, and settled himself at Paris, to continue his studies and prepare himself for future eminence. Shortly after, owing to pecuniary embarrassments of his father, he was compelled to seek remunerative practice, and repaired for this purpose to Olivet, near Orleans, but with the determination to return to Paris at the first opportunity. Two years afterwards a public concourse was held at Paris for several public positions as surgeon; Ricord entered the lists and received the highest honors. Three years afterwards he was appointed surgeon-in-chief to the Hopital du Midi, which position he retained until his retirement in 1860.

Ricord is, perhaps, the most busy physician at present in Paris. His motto is *Ægrotantis animam reconfortare conor.* Rising at seven o'clock, he takes a cup of coffee, and then enters his *coupe* to pay his professional calls. About three o'clock he returns to take his only meal for the day. Then begin his office consultations, which continue without interruption until midnight and sometimes longer.

The beautiful dwelling which he occupies in the *Rue de Tournon* is divided into two distinct portions; at the left are the household apartments, at the right his professional apartments. The latter compose the doctor's study, and five rooms, always full during consultation hours. The first is the common reception-room. It is literally packed with men, each one with a card, upon which is a number telling the order in which he will be attended to. The second apartment is a reception-room for ladies, who ascend by a separate and private stair-case. Into the third apartment are introduced those who require to announce themselves at once, and those who have letters of introduction. The fourth apartment is reserved for the doctor's friends and professional visitors. All these rooms are furnished with curiosities, paintings, statues and other works of art.

The library in the study is surmounted by a gallery of busts of eminent medical men of all times, and with glass cases at the bottom, in which are kept the most beautiful collection of instruments it is possible to see, occupies three sides of the apartment The fourth side is decorated by three portraits, that of Dupuytren in the center, with one of Orfila on the left, and one of Ricord himself on the right. Ricord has received more decorations than any other man in France, except Alexander Dumas.

